# Nanostructured TiO_2_ Surfaces Promote Human Bone Marrow Mesenchymal Stem Cells Differentiation to Osteoblasts

**DOI:** 10.3390/nano6070124

**Published:** 2016-06-24

**Authors:** Marco Vercellino, Gabriele Ceccarelli, Francesco Cristofaro, Martina Balli, Federico Bertoglio, Gianna Bruni, Laura Benedetti, Maria Antonietta Avanzini, Marcello Imbriani, Livia Visai

**Affiliations:** 1 Department of Molecular Medicine, Center for Health Technologies (CHT), UdR INSTM, University of Pavia, Viale Taramelli 3/b, Pavia 27100, Italy; marco.vercellino01@universitadipavia.it (M.V.); francesco.cristofaro01@universitadipavia.it (F.C.); federico.bertoglio01@universitadipavia.it (F.B.); 2 Department of Public Health, Experimental Medicine and Forensic, Human Anatomy Unit, Center of Health Technologies (CHT), University of Pavia, Viale Forlanini 8, Pavia 27100, Italy; gabriele.ceccarelli@unipv.it (G.C.); martina.balli01@universitadipavia.it (M.B.); laura.benedetti@unipv.it (L.B.); marcello.imbriani@unipv.it (M.I.); 3 Department of Chemistry-Physical-Chemistry Section, University of Pavia, Viale Taramelli 16, Pavia 27100, Italy; giovanna.bruni@unipv.it; 4 Laboratory of Transplant Immunology/Cell Factory, Fondazione IRCCS Policlinico “San Matteo”, P.le Golgi 19, Pavia 27100, Italy; ma.avanzini@smatteo.pv.it; 5 Department of Occupational Medicine, Toxicology and Environmental Risks, S. Maugeri Foundation, IRCCS, Via S.Boezio 28, Pavia 27100, Italy; marcello.imbriani@fsm.it

**Keywords:** human bone marrow mesenchymal stem cells, bone tissue engineering and regeneration, nano-patterning modification, titanium dioxide surface, biomedical applications

## Abstract

Micro- and nano-patterning/modification are emerging strategies to improve surfaces properties that may influence critically cells adherence and differentiation. Aim of this work was to study the in vitro biological reactivity of human bone marrow mesenchymal stem cells (hBMSCs) to a nanostructured titanium dioxide (TiO_2_) surface in comparison to a coverglass (Glass) in two different culture conditions: with (osteogenic medium (OM)) and without (proliferative medium (PM)) osteogenic factors. To evaluate cell adhesion, hBMSCs phosphorylated focal adhesion kinase (pFAK) foci were analyzed by confocal laser scanning microscopy (CLSM) at 24 h: the TiO_2_ surface showed a higher number of pFAK foci with respect to Glass. The hBMSCs differentiation to osteoblasts was evaluated in both PM and OM culture conditions by enzyme-linked immunosorbent assay (ELISA), CLSM and real-time quantitative reverse transcription PCR (qRT-PCR) at 28 days. In comparison with Glass, TiO_2_ surface in combination with OM conditions increased the content of extracellular bone proteins, calcium deposition and alkaline phosphatase activity. The qRT-PCR analysis revealed, both in PM and OM, that TiO_2_ surface increased at seven and 28 days the expression of osteogenic genes. All together, these results demonstrate the capability of TiO_2_ nanostructured surface to promote hBMSCs osteoblast differentiation and its potentiality in biomedical applications.

## 1. Introduction

Bone tissue engineering is a strategy to replace autologous or heterologous bone grafts with an artificial material (scaffold) that mimics the bone structure [1,2,3]. A bone graft substitute may follow a series of indication and should share a higher number of properties, such as a good material that allows a structural framework for bone growth and the good stem cell to produce new bone [4,5], good cell attachment and the absence of immune reactions. The scaffold is asked to attract osteoprecursor cells, such as bone marrow stem cells (BMSCs), and to induce them to the osteoblastic phenotype (osteoinduction). The subsequent step is the production of new bone by the newly formed osteoblasts [6,7]. To properly allow this last event, materials should be resorbable. The resorption induced by osteoclasts may be counteracted by the production of new bone. This gradual process takes a duration in the order of weeks/months and should ideally lead to the complete dissolution of the scaffold. An ideal scaffold should also allow the development of a vascularized network to provide the proper irroration of the regenerated tissue [8]. In addition, it should consent to cell adhesion and promote cell growth and differentiation. It should facilitate extracellular matrix regeneration and cell spatial distribution through the scaffold to mimic the physiological bone architecture [9].

As regards to these aspects, surface topography and chemistry take significant influences on the biological performance of biomedical scaffolds [10]. In this context, titanium dioxide (TiO_2_) represents a gold standard in bone tissue engineering, in fact the nanotopography of TiO_2_ raised several important properties, which allow the use of this scaffold to mimic the morphology and the hierarchical organization typical of the extra cellular matrix (ECMs) in bone [11,12]. TiO_2_ nanostructured surface was obtained by the deposition of a supersonic beam of TiO*_x_* clusters. The typical features of this scaffold showed dimensions in the range 1 to 100 nm. In previous studies, the characteristic of this nanostructured surface, such as the porosity and the nanotopography were analyzed and, in addition, a variety of chemical groups and immobilized functional peptides to functionalize the surface for the improvement of cell attachment and proliferation were evaluated [13,14,15].

Bone marrow stromal cells, for more than twenty years, have represented a good source of osteoblast precursor cells [16]. Recent studies have demonstrated the differentiation potential of human BMSC. Under appropriate culture conditions, these human stem cells can differentiate into ligament, tendon [17], muscle [18,19], nerve [20,21], endothelium [22] or hepatic tissue [23]. Moreover, human bone marrow mesenchymal stem cells (hBMSCs) not only contribute structurally to tissue repair but also possess strong immunomodulatory and anti-inflammatory properties that may influence tissue repair by modulation of local environment.

In this study, we evaluated the biocompatibility of Titanium dioxide nanostructured clusters deposited on a coverglass surface (Tethis^®^ company, Milan, Italy), with respect to a microscopy coverglass (Glass). We performed a detailed investigation, in terms of adhesion, proliferation and differentiation towards bone phenotype of human multipotent stem cells on nanostructured TiO_2_ and Glass surfaces. Furthermore, to comprehend the influence of surface nanotopography on hBMSCs adherence and differentiation, the cells were cultivated in the presence (osteogenic medium (OM)) or absence (proliferative medium (PM)) of osteogenic factors. Considering the clinical applications of TiO_2_ nanostructured surface in nanomedicine and bone tissue engineering, the main objective of this manuscript was to elucidate the biological mechanisms of the interaction cell-biomaterial surface, in order to improve the use of surface nanotopography for bone grafts.

## 2. Results

### 2.1. Morphological Evaluation of Nanostructured TiO_*2*_ Surface

**Figure 1:**
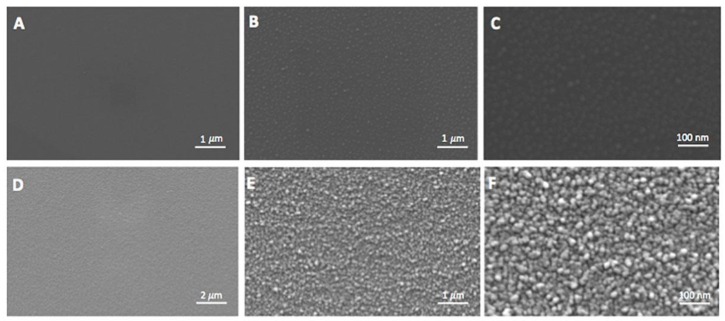
Scanning electron micrographs (SEM) of the Glass surface at: 10,000× (**A**); 50,000× (**B**); and 100,000× (**C**); SEM of the nanostructured TiO_2_ surface at: 10,000× (**D**); 50,000× (**E**); and 100,000× (**F**).

Titanium dioxide surface used in this study was realized by the deposition of a supersonic beam of TiO*_x_* clusters [13]. The surface of the Glass (used as control) and of nanostructured TiO_2_ were different at SEM (Figure 1): a uniform and particulate structure of the clusters, with diameter under 100 nm of dimension was observed for the TiO_2_ surface (Figure 1D–F) but not for the Glass (Figure 1A–C). On the TiO_2_, it is possible to see the typical nanoclusters that begin to be distinguishable at high magnification 50,000× and more evident at 100,000× (Figure 1E,F). Further chemical characterizations were previously reported [13].

### 2.2. Cell Attachment and Cytoskeleton Morphology

**Figure 2:**
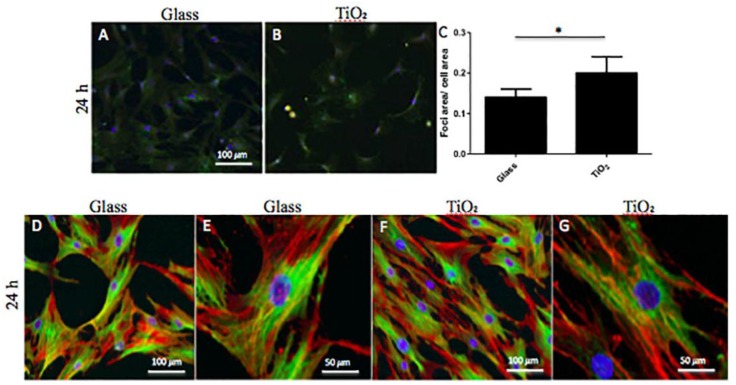
Human bone marrow mesenchymal stem cells (hBMSCs) adhesion and morphology on Glass and nanostructured TiO_2_ surfaces at 24 h. (**A**,**B**) confocal laser scanning microscopy (CLSM) images of focal adhesion for cells seeded on Glass (**A**) and TiO_2_ (**B**): adherent hBMSCs were fixed, permeabilized and immunostained against phosphorylated focal adhesion kinase (pFAK) as indicated in Materials and Methods section. Nuclei were counterstained with Hoechst 33342. (**C**) Graphical Estimation of relative foci per cell on Glass and TiO_2_. Bars represent normalized values from three or more fields at 20×. (*: *p* < 0.05). (**D**–**G**) CLSM images of tubulin (green fluorescence) and actin (red fluorescence) staining of the hBMSCs cytoskeleton seeded on Glass (**D**,**E**) and TiO_2_ (**F**,**G**). Magnifications: 20× (**D**,**F**) and 40× (**E**,**G**) for both surfaces, Glass and TiO_2_. Nuclei were counterstained with Hoechst 33342.

Cell attachment and morphology at short (24 h) and long (seven days) time incubation were properly analyzed (Figure 2). To evaluate cell attachment, hBMSCs were seeded on the different surfaces (Glass and TiO_2_), cultured for 24 h, then fixed and stained with anti-p-FAK (Y397, green fluorescence).

**Figure 3:**
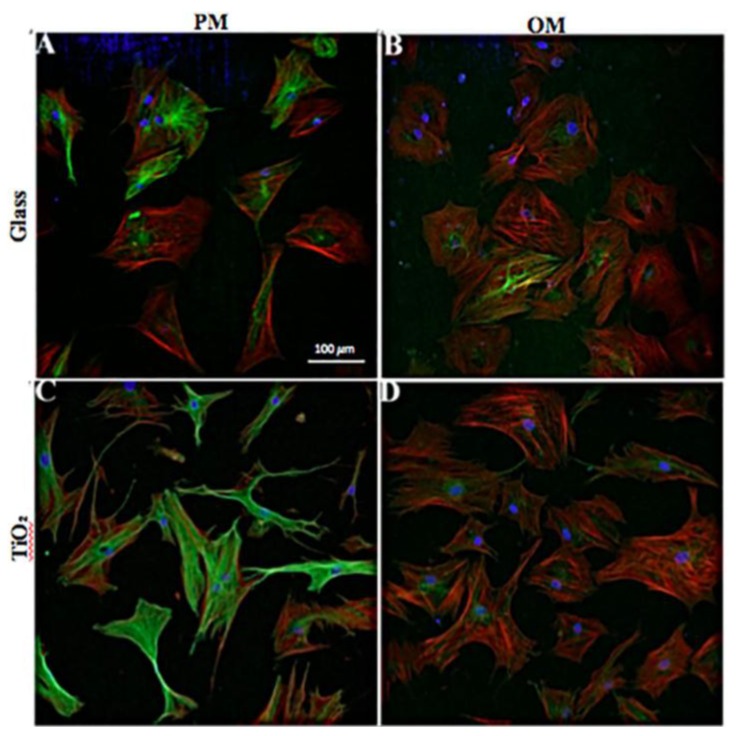
CLSM representative images of hBMSC cells seeded and cultured on Glass and on nanostructured TiO_2_ at seven days: (**A**,**C**) the cytoskeleton of cells cultured in proliferative medium and (**B**,**D**) cells cultured in osteogenic medium on Glass (**A**,**B**) and TiO_2_ (**C**,**D**). Tubulin was stained with goat anti-rabbit Alexa flour 488 antibody, whereas actin was colored in red (Phalloidin). Nuclei were counterstained with Hoechst 33342.

In Figure 2, CLSM images of focal adhesion of cells cultured on Glass (Figure 2A) and TiO_2_ (Figure 2B) are shown representative. From these images, we did not note any visible morphological alterations between cells adherent to Glass and TiO_2_ nanostructured surface. Furthermore, to evaluate possible surface influences, the area of p-FAK positive foci was normalized to cells area: TiO_2_ surface showed an increment of about 5% in foci-positive area (Figure 2C) and the increment was statistical significant (*p* < 0.05). Finally, in order to evaluate the effect of both surfaces on cytoskeleton morphology, cells were observed with CLSM at 24 h and seven days of culture. At 24 h in proliferative conditions, hBMSCs were observed through immunofluorescence of actin and tubulin filaments, as shown in Figure 2D–G. No specific differences in morphology of adherent cells to the different surfaces were observed at 20× and 40× magnification. At seven days, hBMSCs were observed in both PM (Figure 3A,C) and OM (Figure 3B,D) culture conditions. In particular, on Day 7 in proliferative conditions, the cytoskeleton morphology of the seeded cells showed some slight differences. As observed in Figure 3A,C, hBMSC cells cultivated on TiO_2_ showed lamellipodia more prolonged and often divided in two or more branches if compared to Glass surface. This difference was again observed more manifest on TiO_2_ if compared to Glass even if at lower extent in OM culture conditions (Figure 3C,D). The overall shape of the cells remained fibroblast-like. An increment in alpha-tubulin signal was also observed in hBMSCs seeded on TiO_2_ nanostructured surface with respect to Glass (Figure 3D). Notably, it was demonstrated that alpha-tubulin decreased in osteogenic conditions, especially in cells seeded on TiO_2_ surface, with a concomitant increase of actin.

### 2.3. Effects of the Nanostructured TiO_*2*_ Surface on Proliferation and Differentiation of hBMSC Cells to Osteoblasts

**Figure 4:**
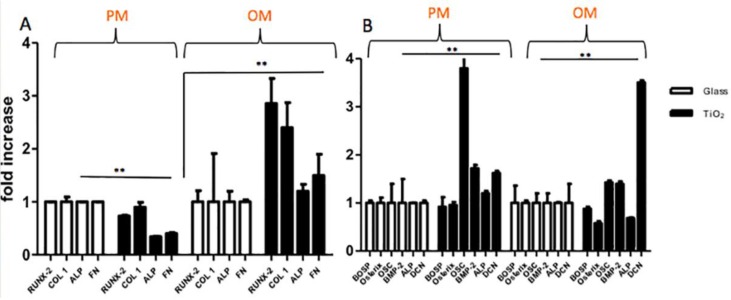
Gene expression of the indicated bone-specific markers as determined by real-time quantitative reverse transcription PCR (qRT-PCR). hBMSCs were seeded and cultured in proliferative medium and osteogenic medium on Glass and TiO_2_ for seven and 28 days, respectively: (**A**,**B**) qRT-PCR were performed on cells cultivated in proliferation medium (PM) and osteogenic medium (OM) for 7 (**A**) and 28 (**B**) days. The graph shows the fold induction of gene expression expressed in arbitrary units setting the expressions of the indicated genes in cells grown in Glass as equal to 1. Statistical significance values are indicated as *: *p* < 0.05, **: *p* < 0.001.

**Figure 5:**
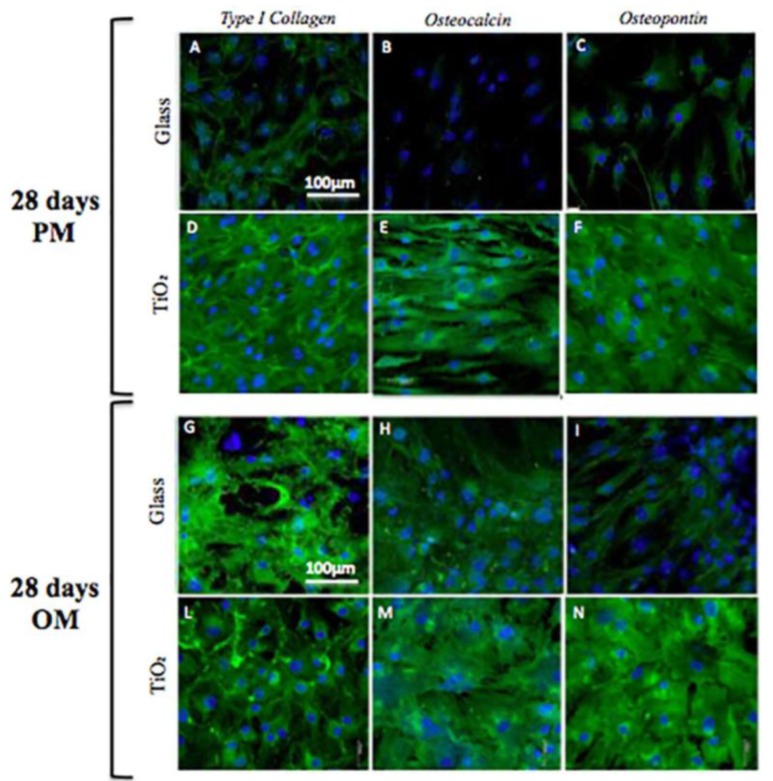
Immunolocalization of Type 1 Collagen (**A**,**D**,**G**,**L**), Osteocalcin (**B**,**E**,**H**,**M**) and Osteopontin (**C**,**F**,**I**,**N**) on Glass (**A**–**C**,**G**–**I**) ) and TiO_2_ (**D**–**F**,**L**–**N**) after 28 days in PM (**A**–**F**) and OM (**G**–**I**,**L**–**N**), respectively. Magnification: 20×; the scale bar shown represents 100 μm. Nuclei (blue) of samples were counterstained with Hoechst 33342.

**Figure 6:**
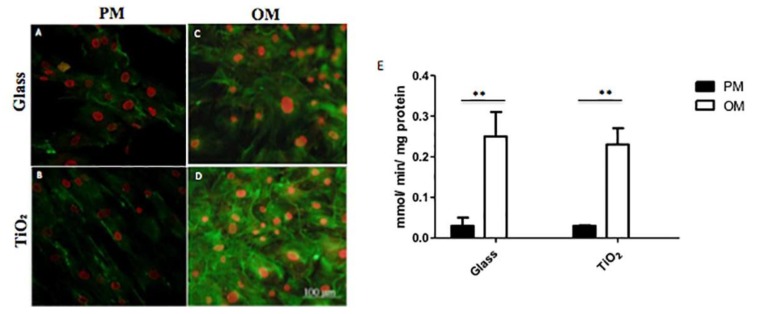
Alkaline phosphatase (ALP) of hBMSC cells seeded onto Glass and TiO_2_ and cultured in proliferative medium (**A**,**B**) or in osteogenic medium (**C**,**D**). (**A**–**D**) Immunolocalization of ALP following incubation with rabbit anti-human ALP primary antibody and detected with goat anti-rabbit secondary antibody (Alexa flour 488). Nuclei (in red) were counterstained with propidium iodide. (**E**) ALP activity determined calorimetrically, corrected for the protein content measured with the bicynchoninic acid (assay) (BCA) Protein Assay Kit and expressed as millimoles of *p*-nitrophenol produced per min per mg of protein. Bars express the mean values ± SEM of results from three experiments in two separated experiments (*: *p* < 0.05; **: *p* < 0.01).

**Figure 7:**
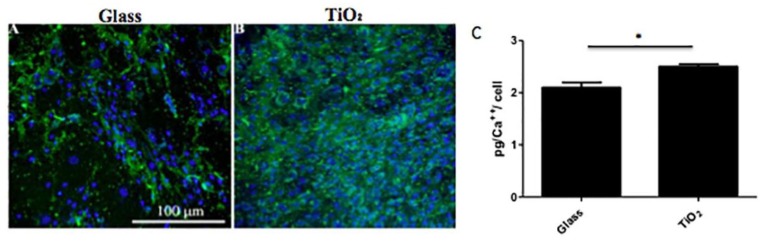
Representative CLSM images at 20× magnification (the scale bar shown represents 100 μm) of calcium deposits from hBM-MSCs cells cultured onto Glass (**A**) or TiO_2_ (**B**). (**C**) Mineralization of extracellular matrix produced by hBM-MSCs cells seeded onto Glass and TiO_2_ as determined by quantification of calcium content. Results are expressed on a per-surfaces basis and are presented as an average ± standard deviation of three measurements in two separated experiments.

To evaluate the effects exerted by Glass and TiO_2_ surfaces on hBMSCs proliferation and differentiation to an osteoblast phenotype, cell viability assay (Alamar blue), apoptosis, gene expression analysis (qRT-PCR, Figure 4), deposition of a calcified bone matrix (ELISA and CLSM, Table 1 and Table 2, Figure 5 and Figure 7) and alkaline phosphatase (ALP) activity (Figure 6) were performed.

#### 2.3.1. Cell Viability

hBMSCs cultured on TiO_2_ nanostructured surface in PM for seven, 14 and 28 days displayed the same viability measurements at each time point with respect to cells seeded on Glass. In fact, at seven days, the values of cell proliferation were 1.45 × 10^5^ ± 0.04 and 1.47 × 10^5^ ± 0.03 for Glass and TiO_2_, respectively. At 14 days, hBMSCs reached the values of 2.1 × 10^5^ ± 0.04 on Glass and 2.5 × 10^5^ ± 0.04 on TiO_2_, with a slight increase in cell number on TiO_2_ surface with respect to Glass. At 28 days, proliferation values were 4.1 × 10^5^ ± 0.04 and 4.61 × 10^5^ ± 0.04 for Glass and TiO_2_, respectively, with always a limited increase for cells cultured on TiO_2_ surface. Nevertheless, no large differences in proliferation were highlighted by Alamar blue assay, and, in addition, no apoptosis was revealed on the two different surfaces (data not shown).

#### 2.3.2. Gene Expression Analyses

In order to characterize cell genotype, gene expression analyses were performed on hBMSC cells cultivated in PM and OM culture conditions on Glass and TiO_2_ surfaces by qRT-PCR technique at seven and 28 days of culture (Figure 4), respectively. In Table 1, the gene primers used for qRT-PCR are reported. Figure 4 shows the fold induction of gene expressed in arbitrary units setting the expressions of the indicated genes in cells grown in Glass as equal to 1. At seven days, gene expression results of ALP, RUNX-2, COL 1 and FN in proliferative and osteogenic culture conditions are presented in Figure 4A. In PM, hBMSCs seeded on TiO_2_ showed no remarkable differences in gene expression of all selected genes that, however, were highlighted in OM. In fact, in OM, the expression of the same genes on TiO_2_ surface were significant higher (between 1.3 and 2 fold higher) with respect to Glass (for RUNX-2 and COL 1 *p* < 0.01, for ALP and FN *p* < 0.05), indicating an osteoinductive effect exerted by the combination of the culture medium with the nanostructured surface. At 28 days, the results of qRT-PCR for BOSP, Osterix, OSC, BMP-2, ALP and DCN genes are shown in Figure 4B. In PM there was an interesting up-regulation of the expression of OSC, BMP-2 and DCN genes in cells seeded on TiO_2_ nanostructured surface with respect to Glass (3 fold higher with *p* < 0.001 for OSC, 0.5 fold higher with *p* < 0.05 for BMP-2 and 0.4 fold higher with *p* < 0.05 for DCN). This genes up-regulation was maintained in OM (Figure 4B), where TiO_2_ nanostructured surface enhanced the expression of OSC, BMP-2 and DCN (0.3 fold higher for OSC, 0.2 fold for BMP-2 and 2.5 fold for DCN, with all *p*-value < 0.01) with respect to Glass. All together, these results confirmed that TiO_2_ nanostructured surface, in comparison to Glass, did not alter gene expression of hBMSC cells; at the same time, in combination with OM conditions, TiO_2_ surface promoted genotype bone differentiation of mesenchymal cells increasing the expression of some key genes involved in osteoblasts development.

#### 2.3.3. Bone Matrix Deposition: Quantification and Immunolocalization Analyses

In order to evaluate the amount of extracellular matrix constituents produced by hBMSCs seeded on Glass and TiO_2_ surfaces, an ELISA assay was performed. In Table 2a,b the protein content results are presented for hBMSCs seeded on the two different surfaces in PM (2a) and OM (2b) at 28 days, as pg/(cells × disk).

There was a significant enhancement of protein deposition for ALP, type-I collagen, type-III collagen, OP, ON, OSC, FN and DCN at 28 days in OM for cells seeded on TiO_2_ nanostructured surface with respect to ECM deposition ratio on Glass (Table 2b). Differently, in PM, there was a slightly greater increase of ECM protein deposition only for FN, ON and OP for cells seeded on TiO_2_ surface with respect to Glass surface (Table 2a). For the other proteins, no peculiar differences were noted. These results are almost in synchrony with gene expression analysis, highlighting the properties of nanostructured surface in combination with OM to direct terminal bone differentiation of hBMSC cells by the translation of specific osteogenic proteins.

At the end of cell culture (28 days), the immunolocalization of bone proteins were evaluated for TiO_2_ and Glass surfaces by CLSM analysis (Figure 5). In particular, immunolocalization of type-I collagen, osteopontin and osteocalcin are reported in Figure 5 (Figure 5A–F for PM, and Figure 5G–N for OM). Immunological studies confirmed that the nanostructured TiO_2_ surface triggered osteogenic differentiation of hBMSC cells both in PM (Figure 5D–F) and OM (Figure 5L–N), with respect to the immunolocalization of osteogenic markers on Glass surface. In fact, the presence of these putative osteogenic proteins, in parallel with previous analyses of gene expression and protein deposition, seemed to be maintained or even slightly potentiated by TiO_2_ surface, especially when combined with OM culture conditions (Figure 5L–N).

### 2.4. Alkaline Phosphatase Activity and Immunolocalization

On Day 28 of culture, ALP activity was measured as reported in Materials and Methods section (Figure 6E). The ALP activity produced per min per mg of protein was higher in osteogenic condition both for cells seeded on Glass and TiO_2_ nanostructured surface with respect to proliferative condition (Figure 6E, *p* < 0.01). Nevertheless, no differences appeared between the two different surfaces in OM.

ALP immunolocalization was then performed using CLSM (Figure 6A–D). Density and morphology of ALP structures were quite similar on Glass both in PM and OM (Figure 6A,C), with no appreciable visible differences. On the contrary, on TiO_2_ nanostructured surface, ALP immunolocalization was greater in OM with respect to PM culture conditions (Figure 6B,D).

### 2.5. Localization of Calcium Deposits

On Day 28 (the end of the culture condition), hBMSC cells cultured in PM and OM on the two different surfaces were observed at CLSM for the detection of calcium deposits after staining with calcein (Figure 7). Cells cultured on Glass (Figure 7A) and on TiO_2_ (Figure 7B) in OM resulted abundant in calcium deposits, even if they were more visible on TiO_2_ nanostructured surfacecompared to Glass. No calcium deposits were observed on either surface when cells were cultured in PM (data not shown). Complexone analysis (Figure 7C) performed on cells cultured in osteogenic conditions revealed similar values between the surfaces, at about 2 pg/cell. However, on TiO_2_ surface, a slightly increase in calcium deposits with respect to Glass surface were observed (Figure 7C, *p* < 0.05).

## 3. Discussion

The main goal of this manuscript was to elucidate, with experimental studies, the biocompatibility of a particular nanostructured surface based on Titanium dioxide deposited on a coverglass (TiO_2_) with respect to a Glass surface. The biocompatibility was investigated in terms of adherence, proliferation and differentiation of hBMSC cells to both types of surface. Bone marrow mesenchymal stem cells have already demonstrated the capacity to spontaneously differentiate into bone on supports/scaffolds [24] with chemical or mechanical stimulation [25,26]. Nevertheless, in this study, we focused on the osteoinductive and proliferative effects of nanostructured TiO_2_ surface.

Nanomaterials represent a particular sub-field of tissue engineering and they have become an innovative technology to be applied in regenerative medicine. Several studies were related to the design of various types of nanostructured scaffolds that could allow the regeneration of different type of tissues [27,28,29]. In addition, it was reported that these materials and matrices have been compared and evaluated for their better biocompatibility and efficacy in supporting the damaged tissue [30,31,32].

Titanium dioxide surface used in this study was produced by the deposition of a supersonic beam of TiO*_x_* clusters (http://www.tethis-lab.com) [13]. This method produced a biocompatible substrate composed by films with a homogeneous nanoscale porosity and roughness [13,14]. This particular nanoscale (with clusters under 100 nm of dimension) may be tuned to modulate specific cell-biomaterial surface interactions. For this reason, we deeply investigated how this nanotopography could interfere with stem cell adhesion, proliferation and differentiation to ameliorate autologous bone grafts. We used human bone marrow mesenchymal stem cells for their plasticity in supporting healing processes and for their large and undeniable use in regenerative medicine.

First of all, we analyzed at 24 h and seven days cell attachment and morphology by performing a careful comparison with cells seeded on TiO_2_ nanostructured surface and Glass (Figure 2 and Figure 3). The evaluation of p-FAK foci indicates unequivocally cellular behavior in response to different matrices [33,34]. In our study, TiO_2_ nanostructured surface promoted in a short time (24 h) the amount of hBMSCs pFAK foci with respect to common Glass (Figure 2). In fact, cells modify focal adhesion in response to modification in extracellular matrix environment, like molecular/biochemical composition or modification in 2D or 3D structure. In this case, the surface of Titanium dioxide changed hBMSCs microenvironment and ECM and provoked a phosphorylation of focal adhesion kinase (FAK) at Y397 that stimulate cell proliferation. As regards morphology, no visible morphological alterations were observed between cells seeded on the two different surfaces, they maintained fibroblast-like morphology, but with an increase of alpha-actin presence and a down-regulation of alpha-tubulin for hBMSCs seeded on TiO_2_ nanostructured surface in OM (Figure 3). These data seems to confirm previous results reported by Khang et al. (2012), showing that the roughness of the modified surface may induce osteoblasts differentiation with an increase of actin expression in cells [35]. The down-regulation of the alpha-tubulin could also be a response of hBMSC cells to the nano-topography of the surface and to the OM that enhanced bone differentiation. In addition, no cell apoptosis was revealed on different surfaces and cell viability analyses at seven, 14 and 28 days both in PM and OM showed no large differences of cell proliferation. We may conclude that the surface of TiO_2_ appeared not toxic for the cells: moreover, these results seems to follow the trends obtained by other groups by culturing cancer cell lines on the same type of biomaterial surface [13].

Secondly, a temporal (at seven and 28 days) and functional pattern of osteogenesis gene expression was analyzed for cells seeded on the two different surfaces (TiO_2_, and Glass). We focused our attention on the expression of different genes at each time point. In particular, at seven days we evaluated the expression of RUNX-2, type-I collagen, ALP and FN, that are genes related to the early and intermediate bone development; in fact RUNX-2 is an important transcription factor associated with osteoblast differentiation [36], and ALP and FN represented early bone formation [37]. Instead, at 28 days, BOSP, Osterix, OSC, BMP-2, ALP and DCN expression were investigated (Figure 4). These genes are osteoblast-specific markers and they are not present in undifferentiated hBMSCs. They could be detected from day 14 in OM, composed by dexamethasone, ascorbic acid, and β-glycerophosphate [38]. Gene expression results confirmed that TiO_2_ surface_,_ in comparison with Glass, did not interfere on gene expression of hBMSC cells. At seven days, the nanostructured surface enhanced osteoblast differentiation by the up-regulation of the genes implicated in early bone development, whereas at 28 days, it promoted genotype bone differentiation by the up-regulation of three important genes, like DCN, BMP-2 and OSC, still in PM and OM (Figure 4b). Surprisingly, TiO_2_ surface induced an overexpression of OSC just in PM, even 3 fold higher with respect to Glass. It was shown by previous studies that, in fact, systemic and local effects of OSC potentially link bone remodeling, vascular calcification, and energy metabolism [39]. Furthermore, the bone-morphogenetic protein 2 (BMP-2) is associated to participate in bone healing and regeneration. Previous studies demonstrated that BMP-2 protein is one of the most potent growth factors that induce mesenchymal stem cell and osteoprogenitor cell differentiation into osteoblasts [40]. Probably, the effect of the TiO_2_ nanostructured surface, in combination with OM, provoked a positive effect on cell bone differentiation by moderate high levels of BMP-2 and an up-regulation of OSC gene.

In order to corroborate gene expression data, we calculated the amounts of extracellular matrix constituents produced by cells at 28 days on different surfaces (Table 2a,b, ELISA assay). For this purpose, we measured the amount of the fundamental bone matrix constituents such as osteopontin, osteocalcin, osteonectin and fibronectin. No appreciable differences in the amounts of proteins in PM conditions were observed, both on Glass and on TiO_2_ nanostructured surface. Otherwise, there was a specific enhancement induced by TiO_2_ surface of osteogenic protein deposition in OM. All these proteins produced by cells represent important markers of bone development: osteopontin is known to play an important role in cell adhesion [41] and calcification of mineralized tissue, whereas DCN represents an osteoblasts terminal differentiation marker [42]. Osteonectin is a calcium and collagen binding ECM glycoprotein and modulates cell-matrix interactions [43]. In summary, based on molecular and protein results, we may assume that the main effect of TiO_2_ nanostructured surface was to facilitate hBMSC cells adherence and, at the same time, to promote protein ECM deposition and, therefore, cell differentiation.

To reinforce molecular and protein results, immunolocalization of osteogenic markers at 28 days confirmed the osteoconductive role of titanium dioxide surface: the presence of type-I collagen, osteopontin and osteocalcin seems to be more evident on TiO_2_ nanostructured surface in OM with respect to Glass (Figure 5). In addition, quantitative analysis of the calcium mineral content showed that TiO_2_ surface promoted deposition of newly mineral matrix (Figure 7), indicating that, more likely, the physical-chemical clusters of the nanosurface promoted Ca^2++^ deposition. Accordingly, to these data, also the increase of ALP activity was consistent to mineral matrix deposition (Figure 6), underlying the mechanism that osteoblastic marker proteins such as alkaline phosphatase and the neo-formed mineralized extracellular matrix (ECM) were a consequence of osteoblastic differentiation supported by the nanostructured material.

In summary, this study should be considered a preliminary in vitro investigation to setup further analyses of the effects of nanostructured surfaces on human multipotent cells and subsequently to be translated to animal and human trials. We demonstrated that the nanostructure of TiO_2_ surface could be successfully employed for in vitro studies of biocompatibility and we speculated that this biomaterial surface might be a good promising surface for bone tissue engineering applications.

## 4. Materials and Methods

### 4.1. Biomaterials

Microscopy coverglass coated with a film of cluster-assembled TiO_2_ were purchased from Tethis (http://www.tethis-lab.com/) [13]. Nanostructured TiO_2_ films (thickness of 50 nm) were fabricated on round glass coverslips (15 mm diameter, 0.13–0.16 mm thickness) by a methods previously described by Carbone et al., using a pulsed microplasma cluster source (PMCS) [13]. The surface morphology of cluster-assembled films was characterized by atomic force microscopy (AFM) and by transmission electron microscopy (TEM) [13].

The slides obtained were sterilized under the UV light of our sterile cabinet for 1–2 h and then left overnight with complete medium to assess sterility. This step lets also the proteins of the serum to attach to the surface. This approach reproduces an in vivo situation in which many proteins will adsorb on the surface until complete colonization by the cells. However, studies with the crude surface were also done. The slides were further characterized with SEM imaging prior to any use, to evaluate the topography and the microstructural configuration. As a control surface, the same microscopy coverglass used for the coating deposition of the film of cluster-assembled TiO_2_, was employed for the study.

### 4.2. Reagents

Unless otherwise specified, all reagents were from Sigma Aldrich (St Louis, MO, USA). Dr. Larry W. Fisher (National Institutes of Health, Bethesda, MD, USA) provided us with the rabbit polyclonal anti type-I collagen, anti-osteopontin, anti-osteocalcin and anti-alkaline phosphatase.

### 4.3. Scanning Electron Microscopy Analysis

We analyzed the microstructural surface of TiO_2_ nanostructured surface with scanning electron microscopy (SEM). The biomaterial surface was treated for SEM analysis with 2.5% (*v/v*) glutaraldehyde solution in 0.1 M sodium cacodylate buffer (pH = 7.2) for 1 h at 4 °C. Then, as previously described, samples coated with gold were observed at 50 and 1000× with a Stereoscan 440 microscope (Leica Miicrosystems, Bensheim, Germany) at 8 kV [44,45].

### 4.4. Cell Culture

The human mesenchymal stem cells (hBMSCs) were expanded from discarded residues (3 mL) of bone marrow harvested for hematopoietic stem cell transplantation, (3 mL) after obtaining written informed consent from the healthy donors.

Successively, hBMSCs were phenotypically analyzed in order to evaluate their mesenchymal properties, according to the International Society for Cellular Therapy [46].

### 4.5. Confocal Laser Scanning Microscopy (CLSM) Analysis

#### 4.5.1. Adhesion and Morphological Analysis

For adhesion (24 h in PM) and morphological analyses (24 h in PM and seven days in PM and OM), paraformaldehyde fixed hBMSC cells on Glass and TiO_2_ were permeabilized with 0.1% Triton X-100 for 10 min at room temperature (RT).

*For focal adhesion*: Cells were further incubated overnight at 4 °C with the primary antibody, rabbit antihuman vinculin clone (hVIN-1, Sigma-Aldrich, Saint Louis, MO, USA) and mouse antihuman pFAK (p397) (Sigma-Aldrich). Finally, samples were stained with Alexa-Fluor-633 goat anti-rabbit and Alexa-Fluor-488 rabbit anti-mouse conjugated secondary antibodies (Invitrogen, Carlsbad, CA, USA) for 1 h at RT each one.

*For morphological studies*: Cells either at 24 h in PM or at seven days in PM and OM, were incubated with phalloidin (Alexa-Fluor-488 phalloidin, Invitrogen) for 20 min and then with anti-tubulin (Alexa-Fluor 633, Invitrogen) for 20 min at RT.

All the previous samples were mounted and nuclei were counterstained with Hoechst (Sigma Aldrich). The images were taken by the TCS SPII confocal microscope (Leica Microsystems, Bensheim, Germany) equipped with a digital image capture system at 20× and 40× magnification. In particular, for quantification of foci, more than 10 images were collected and analyzed with ImageJ (http://rsb.info.nih.gov/ij/; National Institutes of Health, Bethesda, MD, USA) to count positive foci of cells seeded on TiO_2_ and on Glass surfaces in each photographed field.

#### 4.5.2. Immunological Studies

Cells at 28 days of culture on Glass and TiO_2_ surfaces in different medium (PM and OM) were stained with primary antibodies against anti–type-I collagen, anti-osteopontin, anti-osteocalcin, anti-alkaline phosphatase (anti-ALP) at 4 °C ON. We used as secondary antobodies Alexa-Fluor-488 goat anti-rabbit IgG (HþL; Invitrogen). Pictures were taken with confocal microscope (Leica Microsystems, Bensheim, Germany) at 40× magnification.

### 4.6. Cell Viability Assay

To evaluate proliferation of hBMSCs at seven and 14 days on the two different surfaces (Glass and TiO_2_), we used Alamar Blue reagent (Sigma Aldrich). According to manufacturer instructions 1:10 dilution in serum free Dulbecco’s modified Eagle’s medium (DMEM) low glucose was added to cells and left for 3 h at 37 °C in 5% CO_2_. Then, absorbance of 100 μL at 595 nm was read and converted to cell numbers with a conversion curve. The cell viability was also performed at the end of culture conditions (28 days in PM and OM culture conditions).

### 4.7. Apoptosis

Apoptosis of hBMSCs was performed by Annexin V technique as previously described [47]. The Annexin V-FITC Apoptosis Detection Kit (Bender Medsystems, Vienna, Austria) was used according to the manufacturer’s instructions to evaluate apoptosis of hBMSCs seeded and incubated for 24 h at 37 °C on the two different surfaces.

### 4.8. Gene Expression Analyses

Total RNA from hBMSC cells seeded on Glass and TiO_2_ surfaces at 7 and 28 days in PM and OM was extracted and retrotranscribed into cDNA as previously reported [48]. Gene expression analyses were performed by qRT-PCR using oligonucleotide primers exemplified in Table 2. The fold expression of each sample was normalized to the GAPDH housekeeping gene and analyzed in triplicate.

### 4.9. Purified Proteins and Polyclonal Antisera

All the proteins analyzed in this manuscript were purified as described previously [49]. Dr. Larry Fisher (National Institutes of Health, Bethesda, MD, USA) provided us with the rabbit polyclonal anti-human antibodies against type-I and -III collagen, decorin, osteopontin, osteocalcin, osteonectin, and ALP [49].

### 4.10. Bone ECM Proteins Extraction and ELISA Assays

The evaluation of ECM produced by cells seeded on Glass and TiO_2_ in PM and OM culture conditions was performed by an enzyme-linked immunosorbent assay (ELISA) as previously reported [50,51]. The total protein concentration was evaluated with the BCA Protein Assay Kit (Pierce Biotechnology, Inc., Rockford, IL, USA). The total protein concentration was in PM 90.1 ± 7.1 µg/mL for the hBMSCs seeded on Glass and 118.5 ± 8.2 µg/mL for the hBMSCs seeded on TiO_2_ nanostructured surface. In OM, the protein concentration was 85.7 ± 2.5 µg/mL for cells on Glass and 105.0 ± 3.3 µg/mL for cells on TiO_2_ surface.

### 4.11. ALP activity

ALP activity of cells was estimated by a colorimetric assay in PM and OM conditions, as previously reported [49,50].

### 4.12. Calcium deposition

To evaluate the calcium deposition, fluorescent calcein detection and calcium–cresolphthalein complexone methods were performed on hBMSCs seeded on both surfaces as described previously [50,51,52].

#### 4.12.1. Calcein detection

hBMSCs seeded on Glass and TiO_2_ at 28 days of culture were stained with a calcein solution 5 mM in PBS (Invitrogen, Carlsbad, CA, USA) for 30 min at 22 °C. The pictures were taken by a confocal microscope at 40× magnification.

#### 4.12.2. Calcium–Cresolphthalein Complexone Method

In order to evaluate calcium deposition, the calcium–cresolphthalein complexone method was performed on hBMSCs seeded on both surfaces at 28 days in PM and OM conditions as previously reported [49,52].

### 4.13. Statistics

Each experiment reported in the Results Section was done in triplicates and at least in 3 separated experiments. Results are expressed as the mean ± standard deviation. Statistical significance between Glass and TiO_2_ surfaces was evaluated by the one-way analysis of variance (ANOVA) with post hoc Bonferroni test.

## Figures and Tables

**Table 1:** Primers used for qRT-PCR. FW: forward primer; RW: reverse primer.

Genes	FW	RW
ALP	5′ CTA TCC TGG CTC CGT GTC C 3′	5′ AGC CCA GAG ATG CAA TCG 3′
BOSP	5′ GGG CAG TAG TGA CTC ATC CG 3′	5′ TCA GCC TCA GAG TCT TCA TCT TC 3′
RUNX2	5′ ACA GTA GAT GGA CCT CGG GA 3′	5′ ATA CTG GGA TGA GGA ATG CG 3′
OP	5′ GTG ATT TGC TTT TGC CTC CT 3′	5′ GCC ACA GCA TCT GGG TAT TT 3′
COL 1	5′ CAT GTT CAG CTT TGT GGA CC 3′	5′ TTC TGT ACG CAG GTG ATT GG 3′
OSC	5′ AAG AGA CCC AGG CGC TAC CT 3′	5′ AAC TCG TCA CAG TCC GGA TTG 3′
OSTERIX	5′ CTC AGC TCT CTC CAT CTG CC 3′	5′ GGG ACT GGA GCC ATA GTG AA 3′
BMP-2	5′ CCT CCG TGG GGA TAG AAC TT 3′	5′ CAC TGT GCG CAG CTT CC 3′
FN	5′ ACC TCG GTG TTG TAA GGT GG 3′	5′ CCA TAA AGG GCA ACC AAG AG 3′
DCN	5′ ACC CCC TCC TCC TTT CCA CAC C 3′	5′ ACC AGG GAA CCT TTT AAT CCG GGA A 3′
* GAPDH	5′ AGC CTC AAG ATC ATC AGC AAT GCC 3′	5′ TGT GGT CAT GAG TCC TTC CAC GAT 3′

* GAPDH is the housekeeping gene.

**Table:** **(a)**

Protein	Bone ECM Produced by hBM-MSCs Cultured for 28 Days in PM (Protein (pg)/Cell)		
	Glass	TiO_2_	Ratio TiO_2_/Glass
**ALP**	63.59 ± 12.69	55.48 ±13.20	0.9
**COL I**	483.73 ±55.87	446.72 ±42.87	0.92
**COL III**	261.8 ± 4.61	247.92 ± 9.67	1
**DCN**	242.02 ± 30.19	206.37 ± 9.75	0.9
**FN**	27.94 ± 1.69	41.78 ± 5.40	1.5
**OSC**	21.81 ± 0.09	21.59 ± 2.53	1
**ON**	9.14 ± 0.20	9.98 ± 1.95	1.09
**OP**	81.96 ± 6.41	83.94 ± 15.60	1.02

**Table:** **(b)**

Protein	Bone ECM Produced by hBM-MSCs Cultured for 28 Days in OM (Protein (pg)/Cell)		
	Glass	TiO_2_	Ratio TiO_2_/Glass
**ALP**	91.98 ± 2.05	160.30 ± 3.14	1.74
**COL I**	442.07 ±5.87	510.0 ± 3.50	1.15
**COL III**	367.74 ± 10.26	456.28 ± 7.81	1.24
**DCN**	349.88 ± 23.27	440.23 ± 4.31	1.25
**FN**	24.21 ± 0.13	30.88 ± 1.60	1.27
**OSC**	40.32 ± 2.45	92.14 ± 1.29	2.28
**ON**	13.84 ± 0.22	26.56 ± 1.11	1.91
**OP**	117.86 ± 10.01	180.67 ± 3.18	1.53

## Abbreviations

The following abbreviations are used in this manuscript:

ALP	Alkaline Phosphatase
BCA	BiCynchoninic Acid (Assay)
BGP	Beta GliceroPhosphate
BMPs	Bone Morphogenetic Proteins
BOSP	Bone Sialoprotein
BSA	Bovine Serum Albumin
CaP	Calcium Phosphate
CD	Cluster differentiation (receptor)
CFU-F	Colony Forming Units-Fibroblastoid
CLSM	Confocal Laser Scanning Microscope
COL1	Type I collagen
COL3	Type III collagen
DCN	Decorin
DMEM	Dulbecco's Modified Eagle Medium
DNA	Deoxyribonucleic acid
DXM	Dexamethasone
ECM	Extracellular Matrix
EDTA	Ethylene Diamine Tetra acetic Acid
ELISA	Enzyme Linked Immuno Sorbed Assay
FACS	Fluorescence Activated Cell Sorting
FAK	Focal Adhesion Kinase
Fn	Fibronectin
GAPDH	Glyceraldehyde Phosphate Dehydrogenase
hBMSC	Human bone marrow mesenchymal stem cell
mRNA	Messanger RNA
OM	Osteogenic medium
ON	Osteonectin
OP	Osteopontin
OSC	Osteocalcin
Osx	Osterix
PM	Proliferative medium
RNA	Ribonucleic Acid
RUNX2	Runt-related Transcription Factor
